# Shifting responsibilities: A qualitative study of how young people assume responsibility from their parents for self‐management of their chronic kidney disease

**DOI:** 10.1111/hex.13549

**Published:** 2022-06-30

**Authors:** Ruth Nightingale, Gretl A. McHugh, Veronica Swallow, Sue Kirk

**Affiliations:** ^1^ Centre for Outcomes and Experience Research in Children's Health, Illness and Disability (ORCHID) Great Ormond Street Hospital for Children NHS Foundation Trust London UK; ^2^ School of Healthcare, Faculty of Medicine and Health University of Leeds Leeds UK; ^3^ Department of Nursing and Midwifery Sheffield Hallam University Sheffield UK; ^4^ Division of Nursing, Midwifery and Social Work, School of Health Sciences University of Manchester Manchester UK

**Keywords:** child, chronic illness, grounded theory, long‐term condition, parent, qualitative, self‐management

## Abstract

**Introduction:**

The responsibility for managing a long‐term condition (LTC) such as chronic kidney disease (CKD) typically transfers from parent to child, as children become older. However, children can find it challenging to become independent at managing their LTC, and evidence for how healthcare professionals (HCPs) support transfer of responsibility is limited. This study aimed to explore how young people with CKD assume responsibility for managing their condition and the HCP's role during this process.

**Methods:**

Sampling, qualitative data collection and analysis were guided by a constructivist grounded theory approach. Individual and dyadic interviews, and focus groups, were conducted with 16 young people aged 13–17 years with CKD, 13 parents and 20 HCPs.

**Findings:**

A grounded theory, *shifting responsibilities*, was developed that provides new insights into how young people's, parents' and HCPs' constructions of the transfer of responsibility differed. These diverse constructions contributed to multiple uncertainties around the role of HCPs, when the process started and was completed and whether the endpoint of the process was young people's self‐management or young person–parent shared management.

**Conclusion:**

Families would benefit from HCP support over a longer timeframe that integrates assuming self‐management responsibility with gaining independence in other areas of their lives and focuses on young people ‘doing’ self‐management.

**Patient or Public Contribution:**

Patient and public involvement was integrated throughout the study, with young adults with CKD and parents who had a child with CKD actively involved in the study's design and delivery.

## 1 INTRODUCTION

Over the last 50 years, there has been a fourfold increase in the number of families who have a child with a long‐term condition (LTC).^1^ As LTCs have no cure, they are managed by medication and/or treatment/therapies; consequently, self‐management is a significant component of healthcare.^2^ Self‐management has been defined in different ways, but is usually viewed as ‘the individual's ability to manage the symptoms, treatment, physical and psychosocial consequences and lifestyle changes inherent in living with a chronic condition’.^3^ ^(p.178)^ Although children with LTCs are usually dependent on, or share management with their parents, they are expected to assume responsibility for self‐management as they mature.^4^ Different terms, including, ‘shared management’ and ‘responsibility sharing’, are used when describing the transition to self‐management, and emphasize the role played by others, including parents and health‐care professionals (HCPs), in supporting the child to assume responsibility.^5^

In the United Kingdom (UK), healthcare policy focuses on the transition between child and adult health services as the key period for children to assume self‐management responsibility.^6^ However, recently developed guidelines that recommend self‐management tasks for children aged 0–20 years with LTCs suggest that children are on a trajectory of developing self‐management skills from early childhood.^7^ This highlights the uncertainty around the optimum time for supporting children to begin assuming responsibility for managing their LTC.

A recent integrative review of the parent‐to‐child transfer of LTC self‐management responsibility found that there was limited evidence around HCPs' roles and ambivalence around what supported children to assume responsibility, and parents to relinquish control.^8^ Where the literature did explore HCPs' roles, this was predominantly from the perspectives of children and parents, with a noticeable absence of HCPs' perspectives. Due to this lack of clarity, the review suggested a need for greater understanding of the transfer of responsibility from the perspective of all key stakeholders, including children, parents and HCPs.

Existing research exploring the transfer of responsibility has tended to focus on the most prevalent childhood LTCs such as diabetes and asthma.^8^ Due to the uniqueness of treatment regimens, a condition‐specific approach is needed when studying how children assume self‐management responsibility.^9^ Therefore, this study focused on an under‐researched LTC, chronic kidney disease (CKD). CKD is a progressive LTC that can lead to end‐stage kidney disease, which is fatal without renal replacement therapies such as dialysis or kidney transplantation.^10^ Based on the glomerular filtration rate (i.e., the rate at which kidneys filter waste products), CKD can be classified by Stages 1–5. The higher the stage, the more ‘severe’ the CKD and therefore the more complex the treatment regimen required.^11^ In the UK, children with CKD stages 3–5 are treated by specialist renal multidisciplinary teams (MDTs).^12^ Although some self‐management tasks are common across all LTCs, children with CKD have condition‐specific challenges including renal diets, fluid restrictions or targets, and dialysis, which can be either in‐centre or at home. Many aspects of the treatment regimen are delivered outside of hospital, and as a result, children and parents carry out the majority of management tasks, including activities that are complex and demanding.^10^

Supporting children with CKD to assume responsibility for self‐management is critical due to the progressive nature of the condition, and because difficulties engaging in self‐management can lead to renal failure.^13^ However, fewer than 20% of children on dialysis were perceived by HCPs to function autonomously at transfer to adult services,^14^ and higher rates of organ failure are evident among adolescents, compared to young children and adults living with a kidney transplant.^15^ Therefore, for children with CKD, competent self‐management is vital to avoid poor clinical outcomes, and HCPs and parents need effective ways to help children learn self‐management as they move towards adulthood.^16^ The aim of this study was to address this knowledge gap by (1) exploring young peoples', parents' and HCPs' views on the parent‐to‐child transfer of self‐management responsibility for CKD stages 3–5 and (2) develop a theory to explain the processes that occur during the transfer of responsibility.

## 2 METHODS

Charmaz's^17^ constructivist approach to grounded theory was used. The objective of grounded theory is to construct a theory that is ‘grounded’ in the data, and explains a social process.^18^ Constructivist grounded theory acknowledges that theory developed is based on co‐construction, and the researchers' interests and experiences, their relationships with participants and the research context, all influence what is defined as data.^17^

### 2.1 Sampling and recruitment

Purposive sampling was initially used to achieve maximum variation in terms of young people's age, ethnicity, CKD stage, treatment type and self‐management needs. HCPs were also purposively sampled based on their discipline. As the study progressed, theoretical sampling was used to generate data to support the construction of robust categories.^17^ Within time restrictions, sampling continued until all the categories were theoretically saturated.

Participants were recruited from two UK children's kidney units. The inclusion criteria were as follows: (1) young people aged 13–18 years with CKD stages 3–5 who were required to undertake self‐management, (2) parents/carers of each young person (YP) and (3) HCPs from the respective renal MDT. Potential participants were identified by two local clinicians who worked within each renal MDT. These clinicians explained the study and obtained verbal consent for RN to provide potential participants with written information. All participants provided written consent/assent. A total of 49 participants participated in the study. The sample comprised of 16 young people, 13 parents (11 mothers, 1 step‐father, 1 carer) and 20 HCPs (five renal paediatricians, four nurses, four social workers, three clinical psychologists, three play workers, one dietitian). Table 1 provides information about the participating young people.

**Table 1:** Characteristics of the participating young people

Young people's characteristics	Girls (*n* = 9)	Boys (*n* = 7)	Total
Age			
13	1	2	3
14	1	3	4
15	2	1	3
16	4	1	5
17	1	0	1
Ethnicity			
White	4	3	7
South Asian	3	2	5
Black	2	1	3
Other	0	1	1
CKD stage/treatment			
Pre‐emptive transplant	0	3	3
Dialysis	4	3	7
•*In‐centre haemodialysis*	1	3	4
•*Home dialysis*	3	0	3
Transplant	5	1	6

Abbreviation: CKD, chronic kidney disease.

### 2.2 Data collection

Individual interviews are primarily used to generate data in grounded theory studies; however, focus groups are increasingly being used on their own and in combination with interviews.^19^ Both interviews (individual and dyadic) and focus groups were used to generate data in this study. Dyadic interviews are useful in examining how family members co‐construct an understanding of daily life.^20^ Young people and their parents were offered the opportunity to be interviewed together or separately. Focus groups, which generate data through group interaction, were undertaken with HCPs as they had pre‐existing relationships.^21^

Data collection took place between August 2018 and August 2019, either face to face in the hospital setting or family home, or by telephone, based on participants' preferences. Topic guides (one each for young people, parents and HCPs) were initially developed by RN in consultation with authors and patient and public involvement contributors, based on the literature and the authors' research and practice knowledge and experience. Each topic guide explored similar areas including experience of the transfer of responsibility; understanding of self‐management responsibility; and what supports young people to assume, and parents to relinquish, this responsibility. As the study progressed, topic guides were revised as part of theoretical sampling.

A total of 21 semi‐structured individual interviews were conducted with young people (*n* = 7), parents (*n* = 4) and HCPs (*n* = 10) lasting between 24 and 78 min. Dyadic interviews, lasting between 46 and 93 min, were conducted with nine young person–parent dyads. Thirteen HCPs participated in two focus groups. RN collected all data, although one focus group was cofacilitated by V. S. as it included a larger number of participants. With participants' consent, interviews and focus groups were audio‐recorded, transcribed verbatim and anonymized.

### 2.3 Data analysis

Analysis was an iterative, inductive process, which meant that data collection and analysis were conducted concurrently. Initial and focused coding was used alongside constant comparison to identify analytical, theoretical categories.^17^ Memo‐writing and diagramming were critical in establishing each category's properties, visualizing connections between the categories and in theory construction.^22^ An additional approach^23^ was used to examine how the context of the discussion shaped data generation in the dyadic interviews and focus groups. RN led on analysis, with authors meeting regularly to discuss code/category development and data interpretation. NVivo Plus Version 11 was used to support data analysis and management.

### 2.4 Rigour

Denzin and Lincoln's^24^ criteria of credibility, transferability, dependability and confirmability were used to evaluate the rigour of this study. Recruiting from two sites and using both purposive and theoretical sampling achieved variation in the sample and ensured comprehensiveness and credibility of the data generated. Using grounded theory strategies, such as detailed coding and constant comparison, maintained the trustworthiness and authenticity of the data. Strategies to support reflexivity were used throughout the study. This included consideration of how the authors' professional backgrounds (a combination of research expertise in child/adult LTCs, and clinical experience in occupational therapy and child/adult nursing outside of CKD) may have influenced data generation and analysis.

### 2.5 Ethical issues

Approval was obtained from the UK Health Research Authority (Ref: 226365), a National Health Service (NHS) Research Ethics Committee (Ref: 18/YH/0210) and the NHS Trust Research and Development Departments. For young people, age‐appropriate and developmentally appropriate written and verbal information was provided. Participants under 16 years of age provided assent and their parents provided consent for their child's participation. As transcripts were anonymized, data extracts are identified by the type of participant (young person, parent, HCP) and the participants' numerical study identifier (1–20). Additional data are presented in Appendix S1.

## 3 FINDINGS

**Figure 1:**
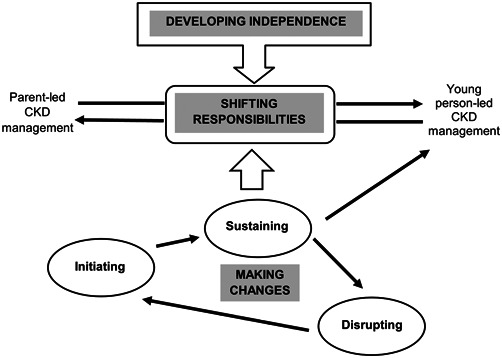
The shifting responsibilities theory. CKD, chronic kidney disease

A grounded theory, *shifting responsibilities*, was constructed from the narratives. The theory is comprised of a core category (*shifting responsibilities*) and two interrelated subcategories (*developing independence* and *making changes*). *Shifting responsibilities* explains the main process that occurs during the parent‐to‐child transfer of self‐management responsibility for CKD. *Developing independence* provides the context for how and when responsibilities shift. The second subcategory, *making changes*, explains how as a result of young people, parents and HCPs adjusting their actions and interactions, the transfer of responsibility was initiated and then either sustained or disrupted (Figure 1).

### 3.1 Shifting responsibilities

Responsibilities moved forwards and backwards along a continuum between parental‐led management and young person‐led management (Figure 1). All the participating young people, regardless of the age when they received their diagnosis, initially experienced self‐management as being parent‐led. Over time, management became increasingly shared and responsibility shifted as the young person took more of the lead in managing their condition. The sense that responsibilities moved along a continuum, where change happened very gradually, yet the extremes were quite distinct, was suggested in one HCP's account:

*What I would like, at an individual pace, was a development while they [child] were growing up. They would be constantly shifting up. If they were acutely unwell, they would step back, then they would continue up again. At one point the parents have no responsibility because they don't live together, and a baby can't do anything. It's going from one extreme to the other*. (HCP6)

Transfer of self‐management responsibility was not a linear process; shifts in responsibility were fluid, bidirectional and uneven, with significant variation between young people. At times, young people transferred responsibility back to their parents, for example, when they felt unwell, were tired or lacked motivation. At other times, parents considered it necessary to assume an increased level of responsibility, such as when their child's condition and treatment changed.

Young people, parents and HCPs tended to have differing views about who should be involved in the transfer of responsibility. All parents believed that it was a key part of their role as a parent to support their child to develop independence, including self‐management. There was a sense that parents saw this as part of their ‘job’:

*You need to support your kids until they're ready to get on their own ladder and support themselves. I'm very supportive with my kids, I like to make sure I've done my job for them, it's what you have to do*. (Parent 5, 16‐year‐old girl)

HCPs believed that they had a responsibility to encourage young people to develop independence in managing their condition; they viewed it as an important aspect of their work in preparing young people for the transition to adult renal services. While some young people and parents welcomed HCPs' help with the transfer of responsibility, others felt ambivalent about whether HCPs should be involved. Only a few young people described how HCPs had supported them, whereas other young people struggled to identify how HCPs had been involved:

*For me, when you say, independent, I think I'm taking my tablets by myself. That's just a habit I got into. We'd tell them [HCPs], ‘She's started taking her tablets by herself’, but I don't remember them having much input. It was a transition that happened at home. I don't know if the doctors have much to do with that really*. (YP1, 14‐year‐old girl)

Different understandings and expectations around the timeframe of the transfer of self‐management responsibility meant that different temporal landscapes existed for young people, parents and HCPs. This included when the process started and when it finished. Some parents started to transfer self‐management responsibility when their child was relatively young in age, especially if they had been diagnosed with CKD at birth or early childhood. In these situations, many parents appeared to take the ‘long view’; they were aware that, in the future, their child would need to develop independence in managing their condition and considered this to be a process occurring over a long period. Both young people and their parents described how they/their child had started to become more involved in self‐management activities while at primary school.

In contrast, the timing of HCP involvement tended to occur later, often after the transfer process had already started, and management responsibility had started to shift. HCPs appeared to view young people assuming self‐management responsibility as part of the transition between child and adult services:

*We owe it to the kids because we owe it to transition. We have to get some independence because otherwise it's one big shock going into an adult service if your parents have done everything*. (HCP3)

As a result, HCPs' involvement in the transfer of responsibility was influenced by UK transition guidelines that recommend that HCPs start planning a young person's transition from child to adult services around the age of 13 years. HCPs' framing of the transfer of responsibility in relation to the provision of healthcare, rather than as a process occurring between a young person and their parents, contributed to the tension around when the transfer of responsibility started and when it finished.

There was uncertainty around whether the endpoint of the transfer process was for the young person to share management with their parent or be ‘fully’ responsible for managing their own condition. None of the young people taking part in the study had reached a point of independently managing their condition, despite some imminently transferring to adult services. Most young people described aiming for self‐management independence and associated this with significant life events such as becoming 18 years old. Parents, however, appeared more ambivalent, with some viewing shared management with their child as a more achievable goal and potentially the endpoint of the transfer process. Although most HCPs aimed for young people to be managing their condition independently by the time they transferred to adult services, some doubted if this was a realistic goal, while others questioned the concept of independence:

*Some of the literature talks about interdependence being a healthier concept than independence. Could we challenge that concept of independence at 18? How many 18‐year‐olds go to university and don't still phone their parents when they get into a pickle? Is there a continuum, a concept of interdependence which needs to shift?* (HCP14)

These tensions around who should be involved, when the process started and ended and knowing when the endpoint had been reached, reinforced the sense that *shifting responsibilities* was a complex, individualized process.

### 3.2 Developing independence

Assuming self‐management responsibility was viewed by young people and parents as a natural extension of the ‘normal’ process where becoming an adult meant attaining independence. Most young people associated increased responsibility with moving towards adulthood. For some, this motivated them to become more involved in self‐management, whereas others perceived that they did not have a choice:

*I've always had to do it. I've always had the help, but as I've got older, I've had to be aware of what I can and can't do. I'm OK with it because I know that it's something that I have to do. I don't have a choice*. (YP8, 16‐year‐old girl)

Young people and parents tended to view the transfer process within a wider context, where assuming self‐management responsibility aligned with developing independence in other areas of their life. This contextual framing meant that some parents viewed their child's engagement in self‐management in terms of ‘typical’ teenage behaviour, especially when they experienced difficulties with transferring responsibility. As a result, parents tended to shift their expectations and made allowances when their child found self‐management difficult:

*She's hit and miss with the tablets. If I say, ‘Have you had your tablets?’ ‘No, it's my life. It's up to me’. You can't push them, but at the end of the day I'm responsible for her. She can be good taking them, she'll have phases. I suppose all teenagers will act this contrary*. (Parent 7, 16‐year‐old girl)

As this account highlights, parents could experience tension around encouraging their child to develop independence while ensuring that their health was maintained. In addition to thinking about their child's future, some parents were hopeful of a future where they themselves would develop independence as their child needed less support with managing their condition. These parents were able to anticipate the impact on their own lives from their child having assumed self‐management responsibility:

*I said to [child], ‘I would like to get on with my life also. You're 18, you're at uni or wherever you want to be. I'm cool with that, but then that gives me permission to get on with my life. I want to be able to do what I want to do. I will always be your mum, you'll always have me, but I feel confident in knowing that you've got this [self‐management] covered’*. (Parent 1, 14‐year‐old girl)

This quotation also illustrates how, despite their child reaching adulthood and developing self‐management independence, many parents perceived that their role as a parent was ongoing. This links to the earlier discussion around when the transfer process ends, and parents' confidence in whether their child would ever be ‘fully’ responsible for managing their condition.

HCPs' knowledge and understanding of child and adolescent development shaped their narratives of working with young people. They recognized that young people were acquiring independence in everyday activities as they became older and described how they discussed these ‘normal’ processes with families:

*We talk about circles of responsibility. When they're a baby, the responsibility all lies with mum and dad. As you get bigger you take on more. You get yourself dressed, you feed yourself, and the logical progression is to take more responsibility for your medicines*. (HCP8)

Despite this recognition, most HCPs viewed self‐management as separate to other activities that young people engaged in. By decontextualizing self‐management, they rarely appeared to support young people with integrating CKD management with daily activities and routines, including how to manage situations when valued activities, such as socializing with friends, and self‐management were in conflict.

The risks associated with developing independence were heightened for young people with CKD. During a dyadic interview, a discussion between a 16‐year‐old, who had recently received a transplanted kidney, and her mother demonstrated their awareness of how CKD impacted on gaining autonomy:

Parent: *We've had conversations where I've said, ‘You aren't ever going to be that young person at uni who gets drunk and wakes up tomorrow afternoon’. There's this element that you have to have control. Life's different for you*.

Young person: *It's always been different. It's not that I used to do that and now I've got to stop and take control. I've always had to have more awareness than my friends. Even if that was not drinking at a party when they were all drinking. I think I'm more responsible than you give me credit fo*r.

Parent: *I do give you credit, but I worry about you hitting that phase that teenagers hit where you go, ‘I want to be the same as everybody else. I'm not going to take my tablets’*. (YP8, Parent 8)

Being aware of the potentially serious consequences of young people assuming responsibility meant that parents and HCPs could experience tensions with encouraging the young person to develop self‐management independence and balancing protection and risk. These tensions around promoting independence while keeping their child safe impacted on parents' motivation to transfer responsibility. Alongside young people's motivation to assume responsibility, this influenced the initiation and continuation of *shifting responsibilities*.

### 3.3 Making changes

Together with *developing independence*, the subcategory *making changes* influenced the process of *shifting responsibilities* (Figure 1). Young people, parents and HCPs made changes to their actions and interactions to initiate and sustain the transfer of responsibility. Alongside the ambiguity around *when* the transfer process started, there was also ambiguity around *how* to initiate this process, and whether to adopt a ‘doing’ or ‘knowing’ approach. Parents' decisions to start transferring responsibility were often based on practicalities, which meant that their initial focus was on their child's ability to undertake a self‐management task safely on their own:

*I used to go to school every two hours to catheterise him. A teacher who felt for me having to come to school, even when it was raining or snowing said, ‘I'm willing to learn what to do’. Somebody from [hospital] went to show her how to do it. Afterwards, I taught him how to do it himself, he was about six when he started doing that*. (Parent 4, 14‐year‐old boy)

In contrast, HCPs' decision that a young person was ready to assume responsibility was based on their demonstration of self‐management knowledge. HCPs appeared more comfortable assessing a young person's knowledge as this was viewed as ‘objective’ in comparison to assessing a young person's skills in performing self‐management:

*I'll start with talking about medication because it's fairly objective. ‘Do you know what medicines you're taking? When do you take them? Do you know what the medicines are for?’*. (HCP9)

Young people, parents and HCPs had similar perceptions around the actions and interactions that sustained the transfer process, including those that promoted a gradual transfer; developed a routine; and facilitated connections with others with CKD. Parents made changes to how they acted and interacted with their child to encourage them to gradually assume self‐management responsibility. One young person's account of how his mother had helped him to learn to change his percutaneous endoscopic gastrostomy (PEG) highlighted one of the techniques used by parents to transfer responsibility incrementally:

*She'll help me do it on my own. Like, when I have to change my PEG, she'll show me how I take it out, and the process of putting it back in. Then the next time I have to do it while she watches*. (YP4, 14‐year‐old boy)

Although HCPs reported that a gradual transfer of responsibility was helpful, their involvement occurred after responsibilities had already started to shift from parents to their child. Due to these misaligned temporal landscapes, there was a perception that HCP input could support a gradual process if it occurred either at an earlier time point or continued after the young person had moved to adult services.

Adjusting actions and interactions to develop a routine was perceived to sustain the transfer of responsibility. Routines supported young people to balance participation in self‐management with other everyday activities and integrate self‐management into their daily life. When an established routine was disrupted, such as during school holidays, young people and parents found it difficult to sustain the transfer process. Young people used their mobile telephones and mobile applications (apps) to create routine by setting alarms to remind them to take medication; diarizing self‐management activities that did not happen daily such as injections; and to monitor their fluid intake. However, as young people found currently available apps of limited use, there was a perception that new tools were needed to support young people to develop routines:

Parent: *We downloaded a water app, it didn't work. If there was an app where you put in your tablets, it bleeped to tell you when to take them, you tick off how much you've drunk, then when its finished, it bleeps and congratulates ‘You've drunk your water today’*.

Young person: *Yes, that would be good. The water app we tried, you could never have four and a half litres on it, it would be one litre. It wouldn't alert you to drink, it just put down what you'd drank out of the litre*. (YP17, Parent 17, 16‐year‐old girl)

Interactions with other people who had CKD were perceived as sustaining the transfer of self‐management responsibility through providing emotional and informational support. During a focus group, HCPs discussed why a meeting between two boys who both needed to self‐catheterize had been beneficial when one of them was not engaging in self‐management:

HCP18: *That was really successful. It was two boys, peer group. The younger one was going to look at the older one and think ‘He's cool, he's dealt with it, so I can deal with it’*.

HCP17: *It's the lived experience. A doctor can tell you the practicalities of what it's going to be like, but no one can tell you what it's really like unless it's someone who's done it*.

Despite the perceived benefits, young people's and parents' connections with others with CKD appeared to be limited. Therefore, young people and parents welcomed increased opportunities to meet peers to provide different and additional support to that offered by HCPs.

Following the initiation of *shifting responsibilities*, the transfer process could be disrupted if young people disengaged from assuming self‐management responsibility. When disruption occurred, trust was lost. One young person, who had experienced a rejection episode of her transplanted kidney due to limited engagement in self‐management, described the impact that this had on her relationship with her parents:

*If there's something wrong with my medication, or I'm not drinking properly, my dad will be, ‘I'm going to give you the water’. He'll check on me. ‘You've got to trust me a little bit’. After my rejection, my dad's constantly nagging me*. (YP14, 16‐year‐old girl)

Following a disruption, young people, parents and HCPs made changes to their actions and interactions to reinitiate the transfer process. For example, parents increased monitoring of their child's self‐management and HCPs discussed their concerns with the family, explored possible solutions and involved different members of the MDT. At times, this would reinitiate the transfer of responsibility; however, for some families where trust had been lost, it was unclear whether the transfer process would recommence or whether the process had come to an end.

## 4 DISCUSSION

This study explored the parent‐to‐child transfer of CKD self‐management responsibility. An emergent theory of *shifting responsibilities* was developed that represents young people's, parents' and HCPs' constructions of the transfer of responsibility, the contextual factors that shaped their constructions and the actions and interactions that initiated, sustained or disrupted the process. By being ‘grounded’ in data, this theory has potential to be further developed and used to design interventions to support the process of *shifting responsibilities*.^25^

One of the key contributions that this study has to offer is greater understanding of how young people, parents and HCPs constructed the transfer of self‐management responsibility. As the first study that had an equal focus on young people's, parents' and HCPs' perspectives, findings suggested that their constructions of the transfer process differed, in relation to: who initiated and was involved in supporting the transfer of responsibility; how the transfer process aligned with young people gaining independence in other areas of their life; how the process was initiated and sustained; when the process was completed; and what the outcome was for young people and parents. As a consequence of these differing constructions, there was ambivalence among young people and parents around the HCP's role in supporting the transfer of responsibility. This finding is significant and has implications for practice.

In parents' constructions of their parental role, they were responsible for supporting their child to develop independence; therefore, most parents initiated the transfer process. This finding supports previous research that suggested that parents either proactively started transferring responsibility for self‐management tasks^26^ or initiated the process in response to external events, such as their child starting secondary school.^27^ When initiating the transfer of responsibility, parents' ‘doing’ approach contrasted with the ‘knowing’ approach adopted by HCPs. Although transition checklists and guidance underline the importance of self‐management ‘behaviours’ (‘doing’ self‐management), their focus is on how HCPs can assess knowledge.^7^, ^28^ This study has highlighted how HCPs could obtain a more accurate and holistic picture of a young person's assumption of responsibility, through observing their actual performance of self‐management activities, rather than relying on young people's demonstrations of knowledge.

Young people and parents tended to construct the transfer of responsibility as an extension of the ‘normal’ process of developing independence that occurs throughout childhood.^29^, ^30^ This aligns with Kieckhefer and Trahms's^4^ framework that emphasizes how parents are promoting shared management from early childhood and the child's developmental stage, rather than a specific age, should guide the process. Although recently developed guidance recognizes that children are involved in condition management from early childhood,^7^ it does not consider how self‐management skills are developed in conjunction with gaining independence in other everyday activities. Therefore, it is recommended that further guidance is developed that recognizes how young people's and parents' motivation to engage in the transfer process is affected by broader, contextual issues.

HCPs believed that it was their role to support young people to assume self‐management responsibility. However, they mostly constructed the transfer process in relation to the transition to adult health services and tended to decontextualize self‐management, viewing it as a separate activity to other activities where young people were developing independence. This finding resonates with existing research that found that HCPs focused on how young people with CKD engaged within the clinical environment, rather than their ‘broader developmental journey into adulthood’.^13^ While the concept of ‘developmentally appropriate healthcare’^31^ suggests that the transfer of responsibility should be considered in relation to a young person's development, rather than as part of transition, the continued focus is on adolescence as the developmental stage when HCPs should support young people to develop self‐management skills.^6^, ^32^ The findings of this study suggest that support from HCPs could potentially be enhanced if HCPs' framing of the transfer process was more closely aligned with how young people and families constructed the transfer of responsibility.

Actions and interactions that sustained the transfer of responsibility included those that promoted a gradual process. Similar findings have been reported in studies with young people with other LTCs, including diabetes, and in the theoretical literature.^33^, ^34^ The findings of this study contribute to the evidence base that suggests that young people with CKD need support with the transition to adulthood over a longer period of time.^13^, ^35^ Therefore, HCP involvement from early childhood to support children to become involved in condition management, and collaboration between HCPs in child and adult renal services to support the transfer of responsibility would be beneficial.^36^

Similar to findings from studies involving young people with other LTCs, the use of mobile phones and apps supported young people to adopt new habits and integrate self‐management activities into their daily routines.^37^, ^38^ The lack of apps that reflect the individualized and complex nature of managing CKD highlights the need for research to develop and test digital technology to support young people to establish routines. Connecting with others who had CKD was found to sustain the transfer process. This supports existing research in other childhood LTCs such as type 1 and 2 diabetes^39^, ^40^ and is in line with NHS guidance that advocates peer support.^2^ Evaluations of peer support for young people with CKD in Canada and the Netherlands found that young people gained informational and emotional support, and their self‐management increased^41^, ^42^; however, as one intervention was online and the other a camp programme, further research is needed to evaluate if similar interventions would be transferable to other countries.

Finally, the findings of this study suggested that there were conflicting understandings around the endpoint of the transfer of responsibility. Young people tended to aim for complete independence in managing their condition.^37^, ^43^ Research in type 1 diabetes and cystic fibrosis has conceptualized the transfer process as complete when a young person is independent in self‐management, and parents have no involvement.^26^, ^44^ In contrast, parents in this study were more ambivalent about the outcome of the transfer process and perceived that they would continue to have some involvement in managing their child's CKD, despite their child having assumed responsibility. Although parent–child shared management tends to be viewed as a ‘bridge to full independence’,^45^ some parents perceived shared management with their child as the endpoint, rather than a transitional stage in the process.^46^ As none of the young people in this current study were independently managing their CKD, research with young adults is needed, to extend understanding of how the move into adult services impacts on the transfer of responsibility, the role of parents and the outcome of the transfer process.

### 4.1 Strengths and limitations of the study

The inclusion of HCPs, alongside young people and parents, and the combination of individual/dyadic interviews and focus groups to generate data assisted with gaining a deeper understanding of the transfer of responsibility. Although dyadic interviews can raise particular ethical and practical challenges, the interactions between young people and parents generated rich data. This suggests that using this method may have facilitated young people's voices ‘by providing them with a supportive, comfortable context within which to take part in research’.^47^ ^(p.662)^ Although a diverse sample participated in the study, reliance on clinicians in the two renal teams for approaching potential study participants may have introduced intentional or unintentional selection bias. Study findings were based on the researcher's analysis and interpretation of young people's, parents' and HCPs' accounts. However, reflexivity and regular discussion with the research team and study advisory group, which included parents who had a child with CKD, ensured rigour.

## 5 CONCLUSION

The is the first study to explore the parent‐to‐child transfer of self‐management responsibility for CKD. New knowledge has been generated including a grounded theory, *shifting responsibilities*, that emphasized how young people's, parents' and HCPs' constructions of the transfer of responsibility differed. These diverse constructions contributed to the uncertainty around the role of HCPs, when the process started and was completed, and whether the endpoint of the process was young people's self‐management or young person–parent shared management. Further research with children aged 2–12 years and young adults with CKD would extend understanding of the transfer of self‐management responsibility and provide opportunities to explore the wider applicability of the theory of *shifting responsibilities*. By identifying what sustained the transfer process, this study has also highlighted the need for research to develop and evaluate interventions that are underpinned by the evidence‐base and theoretical literature and involve young people, parents and HCPs as key stakeholders. Families would benefit from HCP support over a longer timeframe that integrates assuming self‐management responsibility with gaining independence in other areas of their lives, and focuses on young people ‘doing’ self‐management.

## CONFLICT OF INTEREST

The authors declare no conflict of interest.

## ETHICS STATEMENT

Approval was obtained from the UK Health Research Authority (226365), a National Health Service (NHS) Research Ethics Committee (18/YH/0210) and the NHS Trust Research and Development Departments.

## Supporting information

Supplementary information.

## Data Availability

Research data are not shared.

## References

[1] Leeman J , Crandell JL , Lee A , Bai J , Sandelowski M , Knafl K . Family functioning and the well‐being of children with chronic conditions: a meta‐analysis. Res Nurs Health. 2016;39(4):229‐243. 27128982 10.1002/nur.21725

[2] NHS England . The NHS Long Term Plan. 2019. Accessed November 11, 2021. https://www.longtermplan.nhs.uk/

[3] Barlow J , Wright C , Sheasby J , Turner A , Hainsworth J . Self‐management approaches for people with chronic conditions: a review. Patient Educ Couns. 2002;48:177‐187. 12401421 10.1016/s0738-3991(02)00032-0

[4] Kieckhefer GM , Trahms CM . Supporting development of children with chronic conditions: from compliance toward shared management. Pediatr Nurs. 2000;26(4):354‐381. 12026469

[5] Gardener L , Desha L , Bourke‐Taylor H , Ziviani J . Responsibility sharing for adolescents with type 1 diabetes: a scoping review. Chronic Illn . 2020. 10.1177/1742395320959406 32998528

[6] NICE . Transition from children's to adults' services for young people using health or social care services. 2016. Accessed November 20, 2021. https://www.nice.org.uk/guidance/ng43/resources/transition-from-childrens-to-adults-services-for-young-people-using-health-or-social-care-services-pdf-1837451149765

[7] Saxby N , Ford K , Beggs S , Battersby M , Lawn S . Developmentally appropriate supported self‐management for children and young people with chronic conditions: a consensus. Patient Educ Couns. 2020;103:571‐581. 31611128 10.1016/j.pec.2019.09.029

[8] Nightingale R , McHugh G , Kirk S , Swallow V . Supporting children and young people to assume responsibility from their parents for the self‐management of their long‐term condition: an integrative review. Child Care Health Dev. 2019;45(2):175‐188. 30690751 10.1111/cch.12645

[9] Hanna KM , Decker CL . A concept analysis: assuming responsibility for self‐care among adolescents with type 1 diabetes. J Spec Pediatr Nurs. 2010;15(2):99‐110. 20367781 10.1111/j.1744-6155.2009.00218.x PMC2851236

[10] DoH . The National Service Framework for Renal Services. Working for Children and Young People. London, UK: Department of Health; 2006.

[11] DoH . The National Service Framework for Renal Services—Part Two: Chronic Kidney Disease, Acute Renal Failure and End of Life Care. London, UK: Department of Health; 2005.

[12] UK Renal Registry . UK Renal Registry 23rd Annual Report. 2021.

[13] Dallimore DJ , Neukirchinger B , Noyes J . Why is transition between child and adult services a dangerous time for young people with chronic kidney disease? A mixed‐method systematic review. PLoS One. 2018;13(8):e0201098. 30071028 10.1371/journal.pone.0201098 PMC6071995

[14] Bell L . Adolescent dialysis patient transition to adult care: a cross‐sectional survey. Pediatr Nephrol. 2007;22(5):720‐726. 17333004 10.1007/s00467-006-0404-z

[15] Kaboré R , Couchoud C , Macher M‐A , et al. Age‐dependent risk of graft failure in young kidney transplant recipients. Transplantation. 2017;101(6):1327‐1335. 27482961 10.1097/TP.0000000000001372

[16] DoH . Transition: getting it right for young people. Improving the transition of young people with long term conditions from children's to adult health services. London, UK: Department of Health; 2006.

[17] Charmaz K . Constructing Grounded Theory. 2nd ed. Sage; 2014.

[18] Birks M , Mills J . Grounded Theory: a Practical Guide. Sage Publications Ltd; 2011.

[19] Charmaz K , Belgrave LL . Qualitative interviewing and grounded theory analysis. In: Gubrium JF , Holstein JA , Marvasti AB , McKinney KD , eds. The SAGE Handbook of Interview Research: the Complexity of the Craft. 2nd ed. Sage; 2012:347‐366.

[20] Reczek C . Conducting a multi family member interview study. Fam Process. 2014;53(2):318‐335. 24410452 10.1111/famp.12060

[21] Morgan DL . Focus groups and social interaction. In: Gubrium JF , Holstein JA , Marvasti AB , McKinney KD , eds. The SAGE Handbook of Interview Research: the Complexity of the Craft. Sage; 2012:161‐176.

[22] Lempert L . Asking questions of the data: memo writing in the grounded theory tradition. In: Bryant A , Charmaz K , eds. The SAGE Handbook of Grounded Theory. SAGE Publications Ltd.; 2007:245‐264.

[23] Lehoux P , Poland B , Daudelin G . Focus group research and “the patient's view”. Soc Sci Med. 2006;63(8):2091‐2104. 16797811 10.1016/j.socscimed.2006.05.016

[24] Denzin NK , Lincoln YS . Introduction: the discipline and practice of qualitative research. In: Denzin NK , Lincoln YS , eds. The Sage Handbook of Qualitative Research. 4th ed. Sage; 2011:1‐19.

[25] Starks H , Trinidad SB . Choose your method: a comparison ofphenomenology, discourse analysis, and grounded theory. Qual Health Res. 2007;17(10):1372‐1380. 18000076 10.1177/1049732307307031

[26] Williams B , Mukhopadhyay S , Dowell J , Coyle J . From child to adult: an exploration of shifting family roles and responsibilities in managing physiotherapy for cystic fibrosis. Soc Sci Med. 2007;65(10):2135‐2146. 17719160 10.1016/j.socscimed.2007.07.020

[27] Rankin D , Harden J , Barnard K , et al. Barriers and facilitators to taking on diabetes self‐management tasks in pre‐adolescent children with type 1 diabetes: a qualitative study. BMC Endocr Disord. 2018;18(1):71. 30316299 10.1186/s12902-018-0302-y PMC6186043

[28] Sattoe JN , Bal MI , Roelofs PD , Bal R , Miedema HS , van Staa A . Self‐management interventions for young people with chronic conditions: a systematic overview. Patient Educ Couns. 2015;98(6):704‐715. 25819373 10.1016/j.pec.2015.03.004

[29] Karlsson A , Arman M , Wikblad K . Teenagers with type 1 diabetes—a phenomenological study of the transition towards autonomy in self‐management. Int J Nurs Stud. 2008;45(4):562‐570. 17046768 10.1016/j.ijnurstu.2006.08.022

[30] Strand M , Brostrom A , Haugstvedt A . Adolescents' perceptions of the transition process from parental management to self‐management of type 1 diabetes. Scand J Caring Sci. 2019;33(1):128‐135. 30152532 10.1111/scs.12611

[31] Farre A , Wood V , McDonagh JE , Parr JR , Reape D , Rapley T . Health professionals' and managers' definitions of developmentally appropriate healthcare for young people: conceptual dimensions and embedded controversies. Arch Dis Child. 2016;101(7):628‐633. 26945026 10.1136/archdischild-2015-309473 PMC5245734

[32] Colver A , Rapley T , Parr JR , et al. Facilitating transition of young people with long‐term health conditions from children's to adults' healthcare services—implications of a 5‐year research programme. Clin Med. 2020;20(1):74‐80. 10.7861/clinmed.2019-0077 PMC6964170 31941736

[33] Dashiff C , Riley BH , Abdullatif H , Moreland E . Parents' experiences supporting self‐management of middle adolescents with type 1 diabetes mellitus. Pediatr Nurs. 2011;37(6):304‐310. 22256691

[34] Reed‐Knight B , Blount RL , Gilleland J . The transition of health care responsibility from parents to youth diagnosed with chronic illness: a developmental systems perspective. Fam Syst Health. 2014;32(2):219‐234. 24749677 10.1037/fsh0000039

[35] Sattoe JNT , Hilberink SR , Peeters MAC , van Staa A . ‘Skills for growing up’: supporting autonomy in young people with kidney disease. J Ren Care. 2014;40(2):131‐139. 24373148 10.1002/jorc.12046

[36] Crawford K , Wilson C , Low JK , Manias E , Williams A . Transitioning adolescents to adult nephrology care: a systematic review of the experiences of adolescents, parents, and health professionals. Pediatr Nephrol. 2020;35(4):555‐567. 30843110 10.1007/s00467-019-04223-9

[37] Babler E , Strickland CJ . Moving the journey towards independence: adolescents transitioning to successful diabetes self‐management. J Pediatr Nurs. 2015;30(5):648‐660. 26190456 10.1016/j.pedn.2015.06.005 PMC5116197

[38] Meaux JB , Green A , Nelson MK , et al. Transition to self‐management after pediatric heart transplant. Prog Transplant. 2014;24(3):226‐233. 25193722 10.7182/pit2014911

[39] Castensoe‐Seidenfaden P , Teilmann G , Kensing F , Hommel E , Olsen BS , Husted GR . Isolated thoughts and feelings and unsolved concerns: adolescents' and parents' perspectives on living with type 1 diabetes—a qualitative study using visual storytelling. J Clin Nurs. 2017;26(19‐20):3018‐3030. 27865017 10.1111/jocn.13649

[40] Mulvaney SA , Mudasiru E , Schlundt DG , et al. Self‐management in type 2 diabetes: the adolescent perspective. Diabetes Educ. 2008;34(4):674‐682. 18669809 10.1177/0145721708320902 PMC2757076

[41] Nicholas DB , Picone G , Vigneux A , et al. Evaluation of an online peer support network for adolescents with chronic kidney disease. J Technol Human Serv. 2009;27(1):23‐33.

[42] Sattoe JNT , Jedeloo S , van Staa A . Effective peer‐to‐peer support for young people with end‐stage renal disease: a mixed methods evaluation of Camp COOL. BMC Nephrol. 2013;14:279. 24359407 10.1186/1471-2369-14-279 PMC3878094

[43] Buford TA . Transfer of asthma management responsibility from parents to their school‐age children. J Pediatr Nurs. 2004;19(1):3‐12. 14963865 10.1016/j.pedn.2003.09.002

[44] Chilton R , Pires‐Yfantouda R . Understanding adolescent type 1 diabetes self‐management as an adaptive process: a grounded theory approach. Psychol Health. 2015;30(12):1486‐1504. 26084198 10.1080/08870446.2015.1062482

[45] Heath G , Farre A , Shaw K . Parenting a child with chronic illness as they transition into adulthood: a systematic review and thematic synthesis of parents' experiences. Patient Educ Couns. 2017;100(1):76‐92. 27693084 10.1016/j.pec.2016.08.011

[46] Meah A , Callery P , Milnes L , Rogers S . Thinking ‘taller’: sharing responsibility in the everyday lives of children with asthma. J Clin Nurs. 2010;19(13/14):1952‐1959. 19538404 10.1111/j.1365-2702.2008.02767.x

[47] MacLean A , Harden J . Reflections on researching with children using “family group interviews” as part of a qualitative longitudinal study. Int J Child Youth Fam Stud. 5(4.1). 2014:649‐665.

