# Supplementary material for: Shifting responsibilities: A qualitative study of how young people assume responsibility from their parents for self‐management of their chronic kidney disease
Source: Health Expect. 2022 Jun 30;25(4):1919–29. doi: 10.1111/hex.13549 (PMC9327865; doi:10.1111/hex.13549)
Supplement: Supplementary file 1 — Supplementary information. [file HEX-25--s001.docx]

**Appendix 1: Supplementary data**

1. **Shifting responsibilities**

| **Participant identifier** | **Data extract** |
| --- | --- |
| YP8 (16-year-old girl) and Parent8 | **Young person**: *Before my transplant I was responsible for taking my tablets of an evening, and you would just know. You wouldn’t even-,*  **Parent:** *She only took two tablets. She took them at night and at that point I never used to check in. Now and again I used to say, ‘Have you taken your tablets?’ when I said goodnight, but it’s not like it is now. I think it’s the importance of the tablets, because tacrolimus [immunosuppressive medication], if you forget it, it’s massive…. I was a lot more slapdash then.*  **Young person:** *I would like you to get like that again, to be honest.*  **Parent:** *So would I.* |
| YP5 (16-year-old girl) | *It’s OK, some bits are hard though. I’m lazy, so some days I just don’t want to do it. On those days, I’ll do it, but my parents might help with bits.* |
| Parent6 (14-year-old boy) | *We started it at home, me and my husband, but the parents, the nurses, the doctors and even the dietitian, everybody was involved. Everybody was carrying it along and worked as a team with the parents to achieve where we’re at today.* |
| HCP8 | *We take a paternalistic stance as we feel that some 11-year-olds are too little to have those discussions or, sorry, I should say developmentally not at an appropriate stage where the actual discussions of transition would be a bit much for them. But, generally, at an age of 11 to 13, we’ll start to formally go through the process…it’s mainly through the transition process.* |

1. **Developing independence**

| **Participant identifier** | **Data extract** |
| --- | --- |
| YP18 (16-year-old boy) | *When I’m 18 I’m going to uni and I can’t rely on my mum then, I have to remember to do all this stuff [self-management activities] myself. So, I might as well start earlier, start doing it nowadays.* |
| Parent11 (13-year-old boy) | *Because he's had it since he was born we've always done it [manage his CKD]. I've tried to say, ‘Come on,' and never helped him with things. He has to learn to do it because ultimately it's his condition not mine, but at the same time he's still a child so it's tough love versus smothering. Also because your condition’s got worse, you’re realising more what it means when your kidney’s fail. This is helping you so you can work towards being more grown up and taking responsibility.* |
| HCP1 | *Because the child’s health is such a precious commodity, it’s a hard prospect for the parents to let go of it. If the stakes are high, you’ve got a lot to lose, which in the worst case is the loss of a transplant. I say to parents, ‘It’s easy for us to say that [to let go], but you’ve lived through your child being on dialysis, you don’t want it to happen again.’ I’m a parent, I understand parents who struggle to let go, and I often come across people who are in that situation because things have gone desperately wrong for them.* |

1. **Making changes**

| **Participant identifier** | **Data extract** |
| --- | --- |
| HCP6 | *When they become teenagers, I spend my time trying to make them tell me how they are. I ask, ‘Why are we meeting today?’ They look taken aback by that, think that I should know. I persist and say, ‘I know why we’re meeting, but it would be good to hear what you think.’ They say, ‘I’m here for a check-up’. Then I say, ‘What kind of check-up?’ They might say ‘The kidneys’. Sometimes it becomes a bit tense and I say, ‘You do remember? It is like going to school, to come in to me. I always ask you question*s’. |
| YP1 (14-year-old girl) and Parent1 | **Young person:** *It’s always been in my life, I knew that I’d have to take on responsibility at some point. Mum didn’t throw it in my face, ‘Here, you can take them. I’m not going to watch, I’m not going to do anything.’*  **Parent:** *I used to sit her on the kitchen worktop, and we used to count her tablets, ‘Bedtime tablets, morning tablets…’ That went on for a long time. She was, ‘I can take them, Mummy.’ I was, ‘How can you? Do you know?’ I’d supervise this, supervise this, supervise this, and then it was like, ‘Yes, she can take them.*’ |
| Parent5 (16-year-old girl) | *If you look at it [food], and you know that’s going to make you ill, then if you want it, have it within guidance, don’t have loads of it. Every day we take it and see how we progress in the day. We’ll think, ‘That’s gone right,’ or ‘We need to tweak that a little bit’. Eventually you get there, you get your routine and then it’s job done.* |
| YP18 (16-year-old boy) | *It is helpful, because it shows you that you’re not the only one going through this. There are other people with the same condition as you, and you can feel relaxed about it.* |
